# Recent advances in sarcoma therapy: new agents, strategies and predictive biomarkers

**DOI:** 10.1186/s13045-024-01650-6

**Published:** 2024-12-18

**Authors:** Minggui Pan, Maggie Zhou, Lu Xie, Nam Bui, Kristen Ganjoo

**Affiliations:** 1 Department of Medicine Division of Oncology, Sarcoma Program, Stanford University School of Medicine, Stanford, Palo Alto, CA 94305 USA; 2 Musculoskeletal Tumor Center, Peking University People’s Hospital, Beijing, China

## Abstract

Soft tissue and bone sarcomas are a heterogenous group of uncommon mesenchymal tumors with high unmet needs for novel therapeutic and diagnostic strategies. Despite many challenges that persist, innovative therapeutics are emerging. Here we provide a review of the studies presented at the 2024 American Society of Clinical Oncology annual meeting that were focused on sarcoma. There were many outstanding studies that were reported at the meeting. We begin by discussing the clinical studies on soft tissue sarcoma (STS) that included multiple histology subtypes, followed by highlighting developments in cellular therapy, before delving into specific STS histologic subtypes followed by a section covering the studies that were focused on predictive biomarkers. We conclude by discussing the studies in bone sarcomas. Some of the studies discussed here are likely to be practice changing. Some of the early-phase clinical trials have shown encouraging results.

## Soft tissue sarcoma

We start here with discussing the studies on soft tissue sarcoma (STS) that included multiple histologic subtypes. Briefly with the historical context, doxorubicin with or without ifosfamide and gemcitabine with docetaxel regimens remain the front-line therapies for advanced STS [1, 2]. Doxorubicin (60 mg/m^2^) plus trabectedin (1.1 mg/m^2^) versus the standard doxorubicin (75 mg/m^2^) showed improved progression-free survival (PFS) for patients with advanced leiomyosarcoma, however with significantly worse hematologic and other toxicities [3]. Our retrospective study that showed docetaxel and gemcitabine when given every 14 days at gemcitabine dose 1000–1500 mg/m^2^ and docetaxel 50 mg/m^2^ with growth factor support was similarly efficacious compared to the traditional day 1 and day 8 schedule at gemcitabine dose 900 mg/m^2^ and docetaxel 75 mg/m^2^, but with better toxicity profile [4]. Overall, the prognosis for patients with intermediate and high-grade STS remain very poor and our recent study showed it was particularly poor with female patients for causes that remain not understood [5]. Novel treatment agents and strategies are urgently needed to improve clinical outcomes and reduce treatment toxicities.

Grunwald et al. reported the phase 2 MEDISARC trial at the 2023 European Society for Medical Oncology (ESMO) meeting and showed that durvalumab plus tremelimumab versus doxorubicin alone had a trend of improved overall survival (OS) favoring dual checkpoint inhibition in patients with locally advanced unresectable or metastatic intermediate or high-grade STS. At the 2024 ASCO annual meeting, the authors reported that patients treated with dual checkpoint inhibition had better quality of life (QOL) based on the Global Health Score [6]. This study suggests that for some patients with advanced STS, such as those with a histology that is more sensitive to checkpoint inhibitor (undifferentiated pleomorphic sarcoma, dedifferentiated liposarcoma, angiosarcoma, etc.), durvalumab plus tremelimumab combination may be a reasonable option as front-line regimen.

The majority of STS possess cold tumor microenvironments (TME) and show limited responses to immune checkpoint inhibitors [7]. Italiano et al. assessed the combination of the immune checkpoint inhibitor avelumab with the tyrosine kinase inhibitor (TKI) regorafenib in a trial of 49 patients with advanced STS with negative tertiary lymphoid structure within TME (48% leiomyosarcoma, 18% synovial sarcoma, and the rest other STSs). The median OS was 15 months, and 6-month progression-free survival was 22%. They found upregulation of chemokine CXCL10, soluble CD8 antigen, and CD8 + T cell infiltration after the treatment was initiated, however, these changes did not correlate with clinical benefit [8].

In a retrospective study reported by Lee et al. that reviewed 216 patients with advanced sarcoma treated with immune checkpoint blockade, several histologic subtypes were found to have a higher response rate including undifferentiated pleomorphic sarcoma(UPS), angiosarcoma, alveolar soft part sarcoma, Kaposi’s sarcoma and myxofibrosarcoma [9]. Response correlated with high tumor mutation burden (TMB) and PD-L1 expression. These results are largely consistent with the previously reported data [10–13].

Liu et al. reported the single arm phase 2 trial combining doxorubicin and ifosfamide with the anti-PD-1 monoclonal antibody sintilimab as first-line therapy in patients with advanced UPS, synovial sarcoma, myxoid liposarcoma, and dedifferentiated liposarcoma [14]. Sintilimab was given at 200 mg on day 1 with doxorubicin at 60 mg/m^2^ on day 1 and ifosfamide at 1.8 gm/m^2^ on days 1–5 every 3 weeks for 6 cycles, followed by sintilimab maintenance for a total of 2 years or until disease progression. Of 46 patients treated, objective response rate (ORR) was 60%. The response rate was 87% (7/8) for UPS, 65% for synovial sarcoma (13/20), 100% for myxoid liposarcoma (3/3), and 50% for dedifferentiated liposarcoma (5/10). Median PFS was 8.9 months, and median OS was 19.5 months. The response rate reported in this study was quite impressive and the toxicity profile with such a chemo-immunotherapy combination appeared to be not worse than doxorubicin and ifosfamide alone. If this is confirmed in a phase 3 trial, this regimen could potentially be a new standard regimen. Kim et al. reported a trial that combined durvalumab and doxorubicin in 41 doxorubicin-naïve sarcoma patients with ORR of 31.7%, median PFS of 8.2 months, and median OS of 24.1 months. [15] A phase 2 ECOG-ACRIN trial is testing doxorubicin plus pembrolizumab versus doxorubicin alone in patients with advanced UPS, based on a phase 1 data [16].

Zhou et al. reported a phase 1/2 trial with LVGN6051, a 4-1BB agonist monoclonal antibody, in combination with anlotinib, a widely used TKI in China, in patients with advanced STS [17]. A total of 39 patients have been treated, two had a partial response (PR), and several had substantial tumor shrinkage close to a PR.

The Japan Clinical Oncology Group study (JCOG1306) reported the final results that showed improved 5-year OS with AI (doxorubicin 60 mg/m^2^ and ifosfamide 10 gm/m^2^, n = 70) versus DG (gemcitabine 1800 mg/m^2^ and docetaxel 70 mg/m^2^, n = 73) [18]. Chemotherapy was given every 3 weeks for 3 cycles preoperatively and 2 additional cycles postoperatively. At the final analysis with medial follow-up of 6.0 years, the estimated five-year OS was 90.0% for the AI group and 76.1% for the GD group. This reaffirms that AI regimen remains the standard of care in neoadjuvant and adjuvant setting, despite the doxorubicin was lower than the standard used in the United States and Europe. The estimated five-year PFS was 65.2% versus 57.4%. There were no treatment-related deaths. The JCOG1802 trial enrolled 120 patients with advanced STS (leiomyosarcoma n = 31, liposarcomas n = 26, translocation related sarcomas n = 18, other sarcomas n = 45) [19]. The median PFS and OS were 2.9 and 14.8 months for patients treated with trabectedin, 2.2 and 13.3 months for eribulin, and 3.7 and 15.7 months for pazopanib.

Perhaps the most anticipated presentation was SU2C-SARC032, a randomized trial of neoadjuvant radiotherapy and surgery with or without pembrolizumab for patients with advanced UPS or dedifferentiated/pleomorphic liposarcoma [20]. From 2017 to 2023, 143 eligible patients were enrolled, majority of the patients (85%) had UPS and grade 3 histology (65%). Patients were randomized to treatment with standard therapy (neoadjuvant radiotherapy 50 Gy in 25 fractions followed by surgery), or to standard therapy plus pembrolizumab 200 mg every 3 weeks given at the start of neoadjuvant radiotherapy for up to 14 cycles (total of one year). Addition of pembrolizumab improved disease-free survival (DFS) for the entire cohort (HR = 0.57), with estimated 2-year DFS at 72.5% versus 54.5%. Addition of pembrolizumab also improved distant DFS (HR = 0.54). When the analysis was performed separately for patients with grade 2 and 3 histology, it appears patients with grade 3 histology had significant DFS benefit while patients with grade 2 did not show benefit from pembrolizumab. When analysis was performed separately for patients with UPS versus liposarcoma, it appears both cohorts showed significant DFS benefit. There were no differences between the two arms in terms of major surgical complications. Correlative analysis is being performed. This trial strongly suggests significant clinical benefit with addition of pembrolizumab beginning concurrently with neoadjuvant radiotherapy and continuing postoperatively for a total of one year for patients with UPS and pleomorphic/dedifferentiated liposarcoma and opens a new front for investigations in other STS histologic subtypes.

## Cellular therapy for synovial sarcoma and myxoid/round cell liposarcoma

Cellular therapy is entering the solid tumors space with promising results in early and late phase clinical trials. The FDA recently approved Afamitresgene autoleucel (afami-cel), an engineered autologous T-cell receptor (TCR) therapy targeting MAGE-A4, a cell surface protein commonly expressed by synovial sarcoma (SS) and myxoid/round cell liposarcoma (MRCLS). This approval was based on the results from SPEARHEAD-1, a phase 2 trial assessing afami-cel in advanced SS and MRCLS. Eligible adult patients for this therapy must have unresectable or metastatic SS who have received prior chemotherapy, are HLA antigen A*02:01P, -A*02:02P, -A*02:03P, or -A*02:06p-positive, and whose tumor expresses MAGE-A4 antigen [21, 22].

At the 2024 ASCO meeting, D’Angelo et al. reported the planned interim analysis results from the IGNYTE-ESO trial, a phase 2 study assessing the efficacy of letetresgene autoleucel (lete-cel), an autologous engineered T cell receptor therapy that targets the NY-ESO-1 antigen in SS and MRCLS. The IGNYTE-ESO trial met the primary endpoint with an ORR of 40% (18/45) [23]. Approximately 65% of SS and 80–90% of MRCLS express the NY-ESO-1 antigen [24]. Previous phase I studies showed preliminary efficacy supporting the development of lete-cel [25–28]. In the IGNYTE-ESO study, patients were required to be 10 years old or older, HLA-A*02:05, or *02:06 positive, with NY-ESO-1 expression in 30% or more of the tumor. Nine of 23 patients with SS (39%) and nine of 22 patients with MRCLS (41%) achieved a PR. Median duration of response was 10.6 months. The adverse events (AEs) were consistent with those previously reported in early phase studies, with the most common AEs being cytokine release syndrome, cytopenia, and skin rash. This study will likely provide another beneficial cellular therapy for patients with SS or MRCLS.

A separate trial reported by Liu et al. enrolled 8 patients with advanced STS whose tumors expressed NY-ESO-1 and were treated with engineered autologous T cells expressing high-affinity NY-ESO-1-specific TCR [26, 29] Five of the eight patients had SS, 1 patient had myxoid liposarcoma, 1 patient had unspecified liposarcoma, and 1 patient had promyelocytic fibroblastic sarcoma. ORR was 50% (4/8), however with small sample size.

## Angiosarcoma

Taxane and doxorubicin-based chemotherapy have been the standard of care for patients with advanced angiosarcoma for the last three decades [30–32]. Previous attempt to improve the efficacy of taxanes by adding bevacizumab demonstrated no additional clinical benefit [33, 34]. In the 2024 ASCO meeting, there were several abstracts that reported phase 2 clinical trials in patients with advanced angiosarcoma.

Kim et al. presented a phase 2 trial that included 32 patients with unresectable locally advanced and metastatic angiosarcoma, treated with paclitaxel and avelumab. Paclitaxel was given at 80 mg/m [2] on day 1, 8 and 15 every 28 days, and avelumab was given at 10 mg/kg biweekly. The OR) was 50% (15 patients had PR and 1 had CR), median OS was 14.5 months, and PFS was 6.0 months [35].

van Ravensteijn et al. reported a phase 2 trial with 18 patients with advanced angiosarcoma treated with the PD-1 inhibitor cemiplimab at 350 mg/m^2^ every 3 weeks. [36] Best overall response rate at 24 weeks was 27%; 4 patients had PR and 1 had CR. One patient had a PR at 24 weeks and later obtained CR. Three patients with ultraviolet (UV) related angiosarcoma had high TMB and 2 had PR.

The Alliance trial A091902 that compared paclitaxel plus nivolumab (Arm 1, n = 32) versus paclitaxel alone (Arm 2, n = 35) was reported to be a negative trial with no improvement of PFS by the addition of nivolumab; however, patients with face/scalp primary treated with paclitaxel and nivolumab combination showed significant PFS improvement, suggesting that face/scalp primary angiosarcoma may be of distinct biology (such as being more likely to be related to excessive exposure to UV, etc.) [37]. The median PFS was 18.3 and 23.3 months and the ORR was 33% and 34% respectively for arms 1 and 2. There was no significant difference in OS between arms 1 and 2. Arm 3 (cabozantinib plus nivolumab) was reported at the 2023 ASCO meeting by Grilley-Olson et al. with 21 patients treated after progressing on first line taxane. ORR was 62% (13/21) and the ORR was similar regardless of anatomic site [38]. The combination of sunitinib and nivolumab for vascular sarcomas have been previously reported to be an active regimen though it did not reach the specified primary endpoint [39]. These results suggest that taxanes remain an efficacious chemotherapy option, the addition of a checkpoint inhibitor to taxane can be a viable option in some circumstances and that cabozantinib plus nivolumab can be an excellent option as well. It is important to note that SWOG S1609 previously reported an ORR of 25% with ipilimumab plus nivolumab in a 16-patient cohort and that 3 out of 5 patients with face/scalp angiosarcoma obtained a response [40].

## Alveolar soft part sarcoma

Tan et al. reported a study with 29 patients with advanced alveolar soft part sarcoma (ASPS) treated with anlotinib, a widely used TKI in China, and TQB2450, a PD-L1 inhibitor, with an ORR of 79.3% [41]. One notable adverse event was hypertriglyceridemia in 14% of patients. It should be noted that ASPS had been previously demonstrated to be sensitive to immune checkpoint inhibitors which are standard of care for the advanced disease [42, 43].

### Desmoplastic small round cell tumor

Desmoplastic small round cell tumor (DSRCT) is an extremely aggressive sarcoma characterized by the EWSR1-WT1 rearrangement. The prognosis is extremely poor with three-year OS of less than 30%. DSCRT is characterized with a cold TME and low benefit from TKIs [44, 45]. Chemotherapy may initially lead to a brief period of disease response or stabilization, but patients typically experience disease progression quickly. Slotkin et al. profiled more than 200 STS patients for ERBB2 expression and found that some DSRCT appeared to retain ERBB2 expression [46]. They reported 7 patients with DSRCT treated with fam-trastuzumab deruxtecan-nxki (T-Dxd) at 5.4 mg/kg every 21 days. Of the 3 patients evaluated at the time of report, 1 patient had obtained PR, and 2 patients had stable disease (SD). This suggests that for patients whose tumor is positive for ERBB2 expression, T-Dxd may be an option, however, additional data is needed.

## Epithelioid sarcoma

Epithelioid sarcoma (ES) is an aggressive STS, often resistant to chemotherapy and radiotherapy, with limited treatment options [47–49]. It is characterized by the absence of expression of the tumor suppressor gene INI-1 (integrase interactor 1, also known as SMARCB1 and BAF47), a key component of the chromatin remodeling complex SWI/SNF (SWItch/Sucrose Non-Fermentable) [47, 48]. Tazmetostat, an EZH2 inhibitor, was approved for advanced ES based on a basket trial with 62 patients treated, with ORR of 14.5% (9/62), median PFS of 5.5 months and median OS of 19 months. [50] Zhou et al. reported a phase 2 trial of SHR-2554, an oral selective EZH2 inhibitor, in advanced ES. Fourteen patients with advanced ES were treated, 3 obtained a PR, and AEs were mostly expected [51]. The results appeared similar to that with Tazmetostat.

### Gastrointestinal stromal tumor

Li et al. reported the phase 1 trial of NB003, a potent, broad-spectrum TKI that inhibits all KIT and PDGFRA mutations in gastrointestinal stromal tumors (GIST) [52]. There were seven dose levels during the dose escalation phase (3, 6, 12, 20, 30, 35 and 40 mg twice daily). As of January 2024, 42 patients had been treated and evaluated, and confirmed ORR was 26.5%. Responses were durable and seen across a broad spectrum of acquired resistance mutations including mutations in the ATP binding pocket and activation loop of the kinase domain of KIT. There was correlation between responses and ctDNA changes from baseline after treatment. Common AEs included asymptomatic CPK elevation in more than 90% of patients treated. Amylase and lipase elevation was common but largely asymptomatic. Based on the safety and efficacy data of the phase 1 dose escalation trial, the dose expansion cohort was determined to receive 20 mg twice daily.

Schoffski et al. reported the results of StrateGIST 1, a first-in-human, phase 1 study of IDRX-42 in patients with metastatic GIST that had progressed on prior lines of therapy [53]. IDRX-42 (formerly M4205) is a potent and highly selective TKI targeting KIT resistance mutations and was previously shown to have robust anti-tumor activity in xenograft models [54]. The dose escalation trial started with 120 mg daily and escalated to the highest dose of 1200 mg daily. ORR was 23% (15 of 66). Among 14 patients who were treated with IDRX-42, 6 patients obtained a PR (ORR 43%). The response was observed across a broad spectrum of KIT resistance mutations. The dose for the phase 1b dose expansion trial was determined to be 300 mg tablet daily.

Qiu et al. reported updated results from the olverembatinib (HQP1351) trial for patients with succinate dehydrogenase (SDH)-deficient metastatic GIST [55]. The preliminary results of this trial were reported at the previous ESMO and ASCO meetings [56, 57]. The ORR was 23% and clinical benefit rate (CBR) was 92%. A phase 3 trial is being planned. It is important to note that SDH-deficient GIST is resistant to nearly all TKIs with only occasional responses reported [58]. Of note, olverembatinib has been an efficacious TKI for patients with CML with a T315I mutation and was recently added to the NCCN guidelines for CML [57, 59].

Zhang et al. reported the results from a trial assessing the combination of avapritinib and sunitinib for patients who had progressed on multiple lines of therapy [60]. Avapritinib was given at 100–200 mg daily, and sunitinib was given at 25–37.5 mg daily continuously on a 28-day cycle. The safety profile was reported to be acceptable. Four of the 20 patients treated obtained a PR, and the median PFS was 6.6 months. Of note, in the previously reported NAVIGATOR trial, avapritinib was found to have a 17% ORR and response duration of 10.2 months among advanced GIST patients with KIT or non-D842V PGDFRA mutation who received at least three prior lines of therapy, suggesting that avapritinib could be of benefit for some patients who have been previously heavily treated [61].

Wagner et al. presented a poster on Peak trial part 1 results of the phase 3 randomized, open-label, multi-center study of bezuclastinib (CGT9486) plus sunitinib in patients with metastatic GIST who had progressed on imatinib [62]. Patients were given bezuclastinib 300 or 600 mg daily plus sunitinib 37.5 mg daily. Bezuclastinib is a selective TKI that inhibits KIT mutations on exon 9, 11, 17 and 18. Sunitinib is more selective in inhibiting KIT mutation on exon 9, 11, 13 and 14. It was hypothesized that combination of these two TKIs could be more efficacious in patients who have progressed on first-line imatinib as disease develops resistant mutations. Among 19 patients treated, the median PFS of patients treated with both bezuclastinib and sunitinib as second line after progression on imatinib was 19.5 months. For the entire cohort, PFS was 10.2 months with ORR of 27.5%. SARC44 is currently testing bezuclastinib plus sunitinib in a phase 2 trial in patients whose GIST had progressed on sunitinib.

## Rhabdomyosarcoma

Rhabdomyosarcoma (RMS) is a challenging disease to treat and requires multi-modality approaches for best outcomes. Major efforts have been made to improve the outcomes of patients with intermediate and high-risk patients. Several Children’s Oncology Group (COG) trials have failed to show benefit of adding additional drugs on top of the traditional backbone regimen VAC (vincristine, dactinomycin or Adriamycin, and cyclophosphamide) [63–65]. The EpSSG trial which investigated maintenance chemotherapy (vinorelbine 25 mg/m^2^ day 1, 8 and 15, every 28 days and oral cyclophosphamide 25 mg/m^2^ daily for 6 months) after 9 cycles of IVA (ifosfamide, vincristine, dactinomycin with or without doxorubicin) showed improved PFS and OS [66, 67]. COG ARST2031 trial is actively enrolling high-risk RMS patients with aim to compare early use of vinorelbine and maintenance chemotherapy [68]. At the 2024 ASCO meeting, Pappo et al. reported results from the RMS13 trial, a phase 2 trial using risk adapted focal proton beam radiation and/or surgery with the addition of maintenance chemotherapy in intermediate-risk rhabdomyosarcoma [69]. Patients were younger than 22 years old, with embryonal, botryoid, or spine cell RMS, stage 2–3, group 3 disease, or with alveolar, undifferentiated or anaplastic RMS, stage 1–3, group 1–3 disease. Maintenance therapy included four cycles of low dose cyclophosphamide, bevacizumab and sorafenib after 14 cycles of standard VAC. Unfortunately, approximately 25% of patients were unable to tolerate the maintenance therapy. For the entire cohort (n = 45), five-year OS was 75.1%, and five-year event-free survival (EFS) was 67.5%. The outcome of this trial reenforces the need for additional innovative therapies for patients with intermediate and high-risk RMS.

### Desmoid tumor

The phase 3 randomized trial DeFi (Desmoid Fibromatosis) had recently reported results demonstrating superior PFS of the g-secretase inhibitor nirogacestat at 150 mg twice daily compared to placebo which led to its FDA approval in November 2023 [70]. However, ovarian dysfunction on this trial was common, occurring in 27 out of 36 women with childbearing potential, and was a significant AE. Logger et al. reported that 21 out of the 27 patients who developed ovarian dysfunction achieved resolution of the AE. Among these patients11 patients were off nirogacestat with the median time to resolution of 76 days, and the remaining 10 patients who remained on nirogacestat treatment achieved resolution of the AE at a median time to resolution of 171 days. [71] Patient education and close monitoring are critical for treating patients with nirogacestat. A separate abstract reported 29 patients treated during the DeFi trial who had an APC mutation and demonstrated ORR of 38% with nirogacestat versus 13% with placebo [72]. In addition, the nirogacestat-treated patients had greater reduction of tumor size from baseline and volume compared to placebo. These data suggest that nirogacestat may be particularly efficacious for desmoid tumors that harbor a germline APC mutation.

### Tenosynovial giant cell tumor

Tap et al. reported results from the phase 3 MOTION trial with Vimseltinib versus placebo in patients with tenosynovial giant cell tumor (TGCT)) [73, 74]. TGCT is a nonmalignant, locally aggressive tumor affecting the synovium of the joint, bursa and tendon sheath and is caused by upregulation of the CSF1 protein. Vimseltinib is a TKI that inhibits the CSF1 receptor (CSF1R) and was shown to be well tolerated in phase 1/2 trials [75]. Patients were treated with Vimseltinib 30 mg twice weekly or matched placebo. Vimseltinib resulted in 40% ORR and 86% CBR (clinical benefit rate). Vimseltinib-treated patients also showed early and durable functional and symptomatic improvement over placebo. This agent is likely to be approved by the FDA in early 2025. Tap et al. had previously reported results from the phase 3 trial assessing the CSF1R inhibitor pexidartinib for TGCT that led to its FDA approval in August 2019 [76]. ORR with pexidartinib was 39%; however, serious liver enzyme elevations secondary to biliary duct injury were common, necessitating close monitoring.

## Bone sarcomas

### Osteosarcoma

Xie et al. reported the ARTEMIS-002 trial, an open label randomized phase 2 study in patients with metastatic osteosarcoma and other sarcomas that had progressed on standard first line therapy, to evaluated the efficacy of HS-20093, a B7-H3 (B7 homologous 3, also called CD276) directed antibody drug conjugate (ADC) [77, 78]. It was previously shown that expression of B7-H3 correlated with poor prognosis in osteosarcoma [79]. HS-20093 is composed of a fully-humanized anti-B7-H3 monoclonal antibody covalently linked to a topoisomerase 1 inhibitor payload via a cleavable maleimide tetrapeptide linker. The cohort 1 of the study included 42 patients with osteosarcoma that had progressed from standard chemotherapy (16 patients were treated with 8 mg/kg every 3 weeks and 26 patients were treated with 12 mg/kg every 3 weeks), and cohort 2 had 20 patients that consisted of other types of sarcomas (all treated with 12 mg/kg every 3 weeks). Among 23 patients with osteosarcoma treated with HS-20093 at 12 mg/kg, ORR was 17.4% and PFS was not reached at the time of report. PFS was 8.2 months for patients who were treated with HS-20093 at 8 mg/kg but no response was observed. For the 20 other sarcoma patients, ORR was 25% and median PFS was 7.2 months. Responses were seen in 2 patients with Ewing sarcoma, 1 patient with undifferentiated pleomorphic sarcoma of bone, 1 patient with synovial sarcoma and 1 patient with unclassified sarcoma. The study did not detect new safety signals. A phase 3 trial is being planned. Of note, this ADC was also studied in patients with extensive-stage small cell lung cancer and reported at both 2023 and 2024 ASCO meeting with high ORR [78, 80].

Avutu et al. reported the dose finding trial with the Wee1 inhibitor Azenosertinib in combination with gemcitabine in patients with refractory/relapsed osteosarcoma [81]. Thirty-one patients have been treated, and the treatment appears to be well tolerated. PFS at 18-weeks was 39% across all doses (11/28 evaluable patients) which was the primary endpoint. The dose-limiting toxicities included thrombocytopenia and gastrointestinal symptoms that were not unexpected.

### Dedifferentiated chondrosarcoma

Dedifferentiated chondrosarcoma is a very aggressive bone sarcoma, treated clinically with osteosarcoma chemotherapy regimens [82]. Strauss et al. reported results from the dedifferentiated chondrosarcoma cohort in the ImmunoSarc II master trial, a phase 2 trial of sunitinib and nivolumab [83]. Patients were started with an induction phase with sunitinib at 37.5 mg daily for 14 days followed by 25 mg daily in combination with nivolumab at 3 mg/kg until progression. The ORR was 26% (5 of 19), PFS was 5.6 months and 6-month PFS was 46%. Median duration of response was 3.5 months. This combination could be an option for patients with advanced dedifferentiated chondrosarcoma as the current options are extremely limited and outcomes remain extremely poor. This combination was previously tested in a cohort of patients that included osteosarcoma, Ewing sarcoma and chondrosarcoma and met its primary endpoint (more than 30% of patients with PFS at 6-month) [84].

### Ewing sarcoma

SARC037 is a phase 1/2 trial assessing the safety of trabectedin given as a one-hour infusion in combination with low dose irinotecan in patients with relapsed/refractory Ewing sarcoma [85]. The majority of Ewing sarcomas are driven by a fusion gene EWS::FLI1. This fusion gene is neomorphic and possesses prion-like behavior, retargeting and disrupting the BAF complex to maintain oncogenic gene expression programs [86]. Previous data demonstrated little activity of trabectedin administered over 24-h infusion in patients with Ewing sarcoma [87]. There was evidence that trabectedin could reverse EWS::FLI1 transcriptome, and a preclinical model had shown that the combination of trabectedin and low dose irinotecan inhibited Ewing sarcoma cell growth [87–89]. In this study, trabectedin was given at 1 mg/m^2^ over 1-h infusion on day 1, and irinotecan was given at 25 mg/m^2^ on day 2 and 4, every 3 weeks. The confirmed ORR was 28% (5/18), median PFS was 2.9 months, PFS at 6-month was 40% and duration of response was 7.5 months. There was evidence of reversal of EWS::FLI1 transcriptome in the tumors of patients who obtained an ORR. This is an interesting translational study that showed such as a combination could reverse the transcriptome imposed by a potent fusion gene.

### Chordoma

EGFR expression on chordoma cells is well described in the literature [90]. Lipplaa et al. reported a trial with locally advanced or metastatic chordoma patients treated with pan-EGFR inhibitor afatinib at 40 mg daily [91]. From 2018 to 2022, 47 patients were enrolled (four were withdrawn). 34 patients were treated with afatinib as first line, and13 patients were treated as later line. For the 34 patients who were treated with afatinib as first line, the PFS was 12 months, meeting the primary endpoint, while for the 13 patients treated with afatinib in later line, the PFS was only 5 months, failing to reach the primary endpoint. Overall, for the entire cohort, 12-month PFS was 40%, overall median PFS was 8.6 months, and four patients obtained a PR. Dose reduction was needed for 42.6% of patients. Importantly, no improvement on quality of life or pain score was observed. The AEs were common but manageable.

Gounder et al. reported a phase 1 trial of 11 patients treated with a SHP2 inhibitor ERAS-601 as monotherapy or in combination with cetuximab. SHP2 (encoded by *PTPN11*) is an oncogenic protein-tyrosine phosphatase that was found to be a genetic dependency for chordoma by genetic mapping using genome-scale CRISPR screening [92]. Two patients received ERA-601 as monotherapy, and 9 patients received the combination. Out of 9 patients evaluated, 1 obtained a PR and 8 had stable disease (SD). The AEs appeared to be expected. Additional data would be interesting to see as more patients are treated in the trial.

### Predictive biomarkers in sarcoma

Identifying predictive biomarkers for prognostic stratification and therapeutic management remains one of the intense research areas in sarcoma. Shulman et al. reported the LEOPARD study in patients with localized osteosarcoma who had pre-treatment ctDNA burden evaluated prospectively to identify patients with inferior outcomes. They found that baseline detection of ctDNA burden (3% or higher) correlated with worse 2-year EFS compared to patients without detectable ctDNA at baseline (56% versus 88%) [93]. This data may be helpful for implementing clinical trials stratifying risk groups based on baseline ctDNA burden in the future.

The core cohesion subunit STAG2 has been found to be frequently mutated in Ewing sarcoma [94–96]. STAG2 occupies enhancer and PRC2-marked regulatory regions, its loss leads to reprogramming of the oncogenic and neurodevelopmental transcriptomes and causes increased metastatic potential of Ewing sarcoma cells in mouse models [95–98]. Gillani et al. reported the COG study with molecular characterization of patients with localized Ewing sarcoma using tumor samples from 354 patients who participated in the AEWS1031, AEWS0031 and INT-0154 trials [94, 99]. Among the 354 patients, 282 had canonical EWS fusion gene identified, 277 had high-quality p53 and STAG2 mutation data and 169 had high-quality STAG2 immunohistochemistry (IHC) data available. They found that p53 mutation (*P* = 0.04), STAG2 mutation (*p* < 0.0001) or protein loss by IHC (*P* = 0.001), and aneuploidy (*P* = 0.005) were associated with higher cumulative incidence of relapse. These data shall be useful for prognostic stratification in clinical practice and for future clinical trials design. It is worth noting that SARC037 has also collected samples for performing ctDNA analysis and we will await the results [85].

ATRX pathogenic variant is one of the most common genomic alterations in sarcoma [100, 101]. Previously it was shown that ATRX pathogenic variant was not associated with OS of patients with advanced intermediate to high-grade STS while PTEN pathogenic variant was associated with inferior OS [100]. Denu et al. performed IHC of sarcoma microarrays to examine the expression of ATRX in sarcoma and found that loss of ATRX expression was associated with inferior OS in patients with uterine leiomyosarcoma, dedifferentiated liposarcoma, and UPS [102]. They also found that loss of ATRX was associated with immunosuppressive TME characterized by T-cell exhaustion and increased M2 macrophages. One caveat is that the patient cohorts in the study consisted of both localized and metastatic disease [102].

## Conclusion

We have provided a comprehensive review of the sarcoma studies presented in the 2024 ASCO meeting and summarized them in Tables 1 and 2. Some of the advances discussed here will likely be practice changing, like the results from SU2C-SARC032 trial that showed addition of one year of pembrolizumab beginning at the neoadjuvant radiotherapy setting provided significant DFS benefit in patients with UPS as well as patients with pleomorphic/dedifferentiated liposarcoma. The IGNYTE-ESO trial may lead to the eventual approval of this innovative therapy for patients with advanced SS and MRCLS. Some of the early-phase trials have shown encouraging results and will continue to expand the horizon in the quest for improving the outcomes of patients with sarcoma.

**Table 1:** Sarcoma studies presented at the 2024 ASCO annual meeting

Studies and references	Phase	Histology	Line of therapy	New agent or regimen	Comparative regimen	Outcomes
MEDISARC [6]	II	Intermediate and high-grade STS	First line	Durvalumab + Tremelimumab	Doxorubicin alone	Trend towards better OS; Better QOL
Italiano et al. [8]	II	TLS-negative STS	First line	Avelumab + Regorafenib	none	OS 15.1 mos; 6-mos PFS 22%
Liu et al. [14]	II	Intermediate and high-grade STS	First line	Sintilimab + doxorubicin + ifosfomide	none	ORR 60% PFS 8.9 mos OS 19.1 mos
Cho et al. [15]	II	Intermediate and high-grade STS	First line	Durvalumab + doxorubicin	none	ORR 31.7% PFS 8.2 mos OS 24.1 mos
Zhou et al. [17]	I/II	Advanced STS	Anthracycline-refractory	LVGN6051 + Anlotinib	none	PR 5% DCR 86%
JCOG1306 [18]	II/III	High-grade STS	Peri-op chemo	Adriamycin + Ifosfamide	Gem + Docetaxel	OS 90% vs 76.1%
JCOG1802 [19]	II	Advanced STS	Second line	Trabectedin	none	PFS 2.9 mos; OS 14.8 mos
JCOG1802 [19]	II	Advanced STS	Second line	Eribulin	none	PFS 3.7 mos; OS 15.7 mos
SU2C-SARC032 [20]	II	Advanced UPS, DDLPS, PMLPS	Neoadjuvant and adjuvant	Pembrolizumab + RT	RT alone	2-year DFS: 72.5% vs 54.5%
IGNYTE-ESO [23]	II	Advanced SS and MRCLS	NY-ESO-1 positive	Lete-Cel	none	ORR 40%
Liu et al [29]	I	Advanced STS	NY-ESO-1 positive	TAEST16001	none	ORR 50%
Kim et al. [35]	II	Advanced angiosarcoma	First line	Paclitaxel + avelumab	none	ORR 50%
van Ravensteijn [36]	II	Advanced angiosarcoma	First line	Cemiplimab	none	ORR 27%
Alliance 091902 [37]	II	Advanced angiosarcoma	First line	Paclitaxel + Nivolumab	Paclitaxel	Similar PFS and ORR
Tan et al. [41]	II	ASPS	First line	Anlotinib + TQB2450	none	ORR 79%
Slotkin et al. [46]	II	DSRCT	Off label	Fam-trastuzumab deruxtecan	none	1/3 PR
Zhou et al. [51]	II	Epithelioid sarcoma	Second line	SHR-2554	none	ORR 20%
Li et al. [52]	I	GIST	Imatinib-refractory or later line	NB003	none	ORR 26.5%
Schoffski et al. [53]	I	GIST	Later lines	IDRX-42	none	ORR 23%
Qiu et al. [55]	II	GIST	SDH-deficient	Olverembatinib (HQP1351)	none	ORR 23%
Zhang et al. [60]	II	GIST	Later lines	Avapritinib + Sunitinib	none	ORR 20%
Wagner et al [62]	III/lead-in	GIST	Second line	Bezuclastinib + Sunitinib	none	ORR 27.5%
Pappo et al. (RMS13) [69]	II	Rhabdomyosarcoma	Maintenance	VAC + CBS	none	5-yr OS 75%
Tap et al. [74]	III	TGCT	Front line	Vimseltinib	placebo	Early functional improvement
Xie et al. [77]	II	Osteosarcoma	Second line	HS-20093	none	ORR 17.4%
Avutu et al. [81]	I	osteosarcoma	Second line	Azenosertinib	none	18-week PFS: 39%
ImmunoSarc [83]	II	Dedifferentiated chondrosarcoma	Front line	Sunitinib + Nivolumab	none	ORR 26%
SARC037 [85]	I/II	Ewing sarcoma	Later lines	Trabectedin + irinotecan	none	ORR 28%
Lipplaa et al. [91]	II	Chordoma	Front line	Afatinib	none	PFS 8.6 mos

*ORR* objective response rate; *PFS* progression-free survival; *OS* overall survival; *STS* soft tissue sarcoma; *TLS* tertiary lymphoid structure; *Mos* months; *DCR* disease control rate; *UPS* undifferentiated pleomorphic sarcoma; *DDLPS* dedifferentiated liposarcoma; *PMLPS* pleomorphic liposarcoma; *DFS* disease-free survival; *SS* synovial sarcoma; *MRCLS* myxoid/round cell liposarcoma; *ASPS* alveolar soft part sarcoma; *PR* partial response; *CBS* cyclophosphamide, bevacizumab and sorafenib; *TGCT* tenosynovial giant cell tumor; *GIST* gastrointestinal stromal tumor; *DSRCT* desmoplastic small round cell tumor

**Table 2:** Predictive biomarker studies presented at the 2024 ASCO meeting

Studies and references	Histology	Biomarkers	Outcomes
LEOPARD [93]	Localized osteosarcoma	Detection of ctDNA	Worse 2-year EFS (56 vs 88%)
Gillani et al. [99]	Localized Ewing sarcoma	Loss of STAG2 expression	Higher rate of relapse
Denu et al. [102]	uLMS, DDLPS, UPS	Loss of ATRX expression	Worse OS

*EFS* event-free survival; *uLMS* uterine leiomyosarcoma

## Data Availability

No datasets were generated or analysed during the current study.
